# Use of Evidence-Informed Deliberative Processes – Learning by Doing: Comment on "Use of Evidence-informed Deliberative Processes by Health Technology Assessment Agencies Around the Globe"

**DOI:** 10.15171/ijhpm.2019.116

**Published:** 2019-12-03

**Authors:** Anthony J. Culyer

**Affiliations:** Centre for Health Economics and Department of Economics & Related Studies, University of York, York, UK.

**Keywords:** Health Technology Assessment (HTA), Evidence-Informed Deliberative Processes, Decision-Making, Cost-Effectiveness, Deliberation

## Abstract

The article by Oortwijn, Jansen, and Baltussen (OJB) is much more important than it appears because, in the absence of any good general theory of "evidence-informed deliberative processes" (EDP) and limited evidence of how they might be shaped and work in institutionalising health technology assessment (HTA), the best approach seems to be to accumulate the experience of a variety of countries, preferably systematically, from which some general principles might subsequently be inferred. This comment reinforces their arguments and provides a further example.

The article by Oortwijn, Jansen and Baltussen (henceforth OJB)^1^ has modest ambitions but is much more important than this might imply. The ambition was to sample health technology assessment (HTA) agencies around the world and ask them to describe how they used “evidence-informed deliberative processes” (EDP) for decision-making in their countries. OJB’s emphasis is less on evidence information and much more on the processes, which seems appropriate given the current imbalance which they report in the literature. They are unnecessarily apologetic about the representative nature of their sample since the aim is neither to test a hypothesis nor to record the prevalence of the various constituents of “process” but rather to provide information and examples drawn from long-established agencies (relatively speaking) and recently established ones. A major finding is that the majority seem to be comfortable with the cost-effectiveness (CE) elements of HTA, where there is a large literature on the theory and methods for applying it, but that they are somewhat at sea with the design and implementation of processes of EDP, where there is no literature of anything like the extent and quality of that on CE.

My first comment is on the introduction of yet another term for deliberative processes – “EDP.” I have elsewhere^2^ ridiculed the needless proliferation of terms for “CE analysis.” The term “deliberative process” has been well-defined for years as an approach “intended to improve the quality of decision-making by allowing for mutual decision-making based on facts.”^3^ It is borrowed from previous concepts of deliberative democracy [eg, Elster^4^] which used the term “deliberative methods.” In fact their neologism brings out what I now think to be a defect in the former definition, with its focus on “fact” or “evidence.” Deliberation is indeed a useful way of teasing out and interpreting evidence but it is no less useful at teasing out and interpreting matters of *value* . So to circumscribe it is both unnecessary and misleading.

Secondly, I do not share their relative confidence with the availability of CE. My own impression is that while most countries will have at least a handful of academic experts who are entirely competent in CE, they are (*a*) only a handful, and (*b*) usually rather remote from the real world of practical use of CE in making public decisions. The staffing of ministries and arm’s length agencies in most low- and middle-income countries (LMICs) is also characteristically weak on the technical side of CE, which makes them less than fully competent commissioners of research evidence, evaluators of the quality of research evidence, and interpreters of evidence generated outside their country. The perception that little guidance is required on the technical side is not therefore a very good guide to what may in fact be a significant weakness wherever there is no established technical tradition in academia or the training of civil servants. Training programmes are required, especially in LMICs – perhaps on a regional rather than a national basis – which as much as possible use the skills of the “handful” locally. The model of sending bright young potential analysts abroad at public (or donor) expense, usually to rich countries, often fails completely as a way of developing local capacity, since many make their subsequent careers either abroad or in the private sector.

The process element is undoubtedly a challenge. This concerns both institutionalisation – the creation of institutions to do the work (or some element of it) and maintenance – supporting ongoing programmes of work and learning from doing what works best. Understanding how best to make arrangements (that are cost-effective) immediately takes one to a highly complex academic and professional crossroads of behavioural science (to predict, for example, the likely behaviour of all the many stakeholder groups involved and affected by the decisions in question); governance (to predict, for example, the consequences of having, or not having, political accountability, advisory versus decisive powers, public participation in decision-making, a degree of independence from political and professional “authorities,” appeals mechanisms, security against conflicts of interest, and like mechanisms); political philosophy (to assess, for example, the desirability of independence, the delegation by ministers of important public decisions, the desirability for its own sake of transparency); political science (to anticipate, for example, political hazards, to engage with external agents like universities, medical colleges and regulatory authorities); the law (to ensure, for example, that all structures are consistent with the constitution and all processes are in line with statutory obligations and natural justice); administrative theory (to understand for example, committee structures that are best suited to the circumstances, committee decision rules like simple majority voting or powers of veto, skills required of committee chairs); industrial economics (to optimise relationships with pharmaceutical and other manufacturers and their national associations, investigate pricing strategies and their consequences for innovation and domestic industry); and communications (to learn, for example, how best to communicate both processes and decisions to the clinical professions, health service managers, patient advocacy groups and, of course, the general public).

This lattice of disciplines and professions militates against there being any single unifying “theory of deliberative processes” so one needs to add other requirement: imagination and descriptive evidence. The design and execution of deliberative processes requires imaginative work by people well-grounded in the practical realities of their own culture and politics^ [1]^ and a systematic accretion of descriptive material from which, over time, one may be able to infer some general principles.

**An Example – the Early Days of NICE** ^5^

The National Institute for Health and Care Excellence (NICE) was created in 1999 to provide authoritative advice for supporting the introduction (and continuation) of clinical governance^ [2]^ in the National Health Service (NHS) in England and Wales. It followed a period in which there had been publicised professional scandals in hospitals and considerable resultant weakening of popular trust in the medical profession – and, for that matter – in “experts” generally. NICE was a form of direct democracy, with substantive decision being delegated to it by Ministers, much consultation and collaboration with those identified as stakeholders^7^ and lots of volunteer human resources. There were no real precedents on which the designers of NICE could draw; they were sensitive to the politics of the day and made guesses as to what might work. The secretary of state of the day (Frank Dobson) said, when asked whether he thought NICE would work, “possibly not but it’s worth a bloody good try.”

NICE sought to be a model of a deliberative process in a number of ways. These were mostly ad hoc, with the founders drawing on their own personal and professional experience:

- There would be open Board meetings to take place bi-monthly around the regions in England and Wales, accompanied by public receptions and ‘Question and Answer’ sessions with the chair.
- Minutes would be published on the NICE web pages before confirmation by the Board.
- The chair and others ensured that opposition parties were fully informed about NICE’s processes and current activity, to avoid NICE being too strongly associated with one political party.
- There was a Partners’ Council. This met once a year to review NICE’s annual report. In the early days it was a source of advice and a forum for exchanging ideas and developing the future plans for NICE. Its membership included representatives from organizations with a special interest in its work such as patient groups, health professionals, NHS management, quality organizations, industry, and trade unions. Members were appointed by the Secretary of State for Health (English minister) and the Welsh Assembly Government. It was abolished after a few years having served a useful function in getting NICE respectably off the ground.
- There would be a Patient Involvement Unit to advise NICE on patient and caregiver involvement, identify patient and caregiver organizations interested in contributing to its work programme, and to promote patient and caregiver contributions by offering training and support for lay people, patients, caregivers and their organizations contributing to the NICE work programme.
- There would be a Citizens’ Council. This was a form of ‘citizens’ jury’ that considered socially value-laden matters referred to it by the NICE Board. Its 30 members had no economic involvement in the health care system and were selected to be representative of the regions and demographic characteristics of England and Wales. Members were paid modestly per day plus travel and subsistence expenses. It met twice a year, adopted a deliberative approach and could call witnesses and commission papers. It was managed, for independence, at arm’s length from NICE by a company specializing in research and community consultation.
- The membership of the Technology Appraisals Committee was to be set broadly. The Committee was to be a standing advisory committee of the Institute, which had a very public profile since it was the source of NICE’s recommendations for the NHS. Members (unpaid) would be appointed for three-year terms (overlapping). They were drawn from the NHS, patient and care-giving organizations, relevant academic disciplines and the pharmaceutical and medical devices industries. Names of Appraisal Committee members were posted on the Institute’s website.
- There would extensive consultation exercises throughout the appraisals process, notably with manufacturers of the technologies under investigation and their comparators.There was to be an appeals procedure. There were to be three grounds for appeal: that the Institute had failed to act fairly and in accordance with the Appraisal Procedure set out in its Guidance to Manufacturers and Sponsors; that it had prepared Guidance which was perverse in the light of the evidence submitted; and that it had exceeded its legal powers.
- There would consultative processes about process. For example, the process through which the procedures for HTA were developed involved several committees with representation of experts from a variety of stakeholders. The outcome was a public document describing the process, who may play what role, opportunities for consultation, etc^8^ there would be extensive liaisons with the eleven Royal Colleges, seven Independent Academic Centres and seven National Collaborating Centres (formed by consortia of the Royal Colleges). The Independent Academic Centres would do most of the literature reviewing, summarising and model re-estimating. They were the Health Economics Research Unit and Health Services Research Unit, University of Aberdeen; the Liverpool Reviews & Implementation Group, University of Liverpool; the Centre for Reviews and Dissemination, University of York; the Peninsula Technology Assessment Group, Universities of Exeter and Plymouth; the School of Health and Related Research, University of Sheffield; the Southampton Health Technology Assessment Centre, University of Southampton; and the West Midlands HTA Collaboration, University of Birmingham.
- NICE created the National Collaborating Centres within consortia of the royal colleges, professional bodies, and patient/carer organizations for developing clinical guidelines. They were: National Collaborating Centres for Acute Care, Cancer, Chronic Conditions, Mental Health, Nursing and Supportive Care, Primary Care, and Women and Children’s Health.
- There would be considerable joint working with NHS Research and Development and the National Coordinating Centre for HTA. It coordinated the national HTA research programme on behalf of NHS Research and Development.

Thus, it was determined that the process of technology appraisal was to be open, multi-disciplinary, multi-professional and multi-institutional, and it would have “lay” participation. It was heavily dependent upon people’s willingness to serve pro bono. It was plain from the outset that very large numbers of people would be involved and the Institute itself would be largely a virtual organization. Few LMICs might be able to afford anything as comprehensive in scale and scope as NICE’s forms of deliberation. NICE itself had to modify some processes on grounds of cost. However, some approximations might be usefully attempted and then developed as experience teaches.

In the absence of a theory of processes, we need to encourage imaginative innovation and much sharing of experience. OJB have given us a good start. It would be great if it were followed up, as experience accumulates, and if more detailed cases could be examined, with the challenges that were faced and with what success resolved and, perhaps, a differentiation of issues that are met everywhere and those that are specific and which may have little to offer outside the particular context. In such a way some general principles might eventually be inferred.

## Ethical issues

Not applicable.

## Competing interests

Author declares that he has no competing interests.

## Author’s contribution

AJC is the single author of the paper.

## Endnote


[1] For a failed attempt to graft a citizens’ council on to an HTA process, see Dobrow et al.^6^



[2] A framework through which NHS organisations are accountable for continually improving the quality of their services and safeguarding high standards of care by creating an environment and local management for accountability and audit of good clinical practice.

